# The Exploding Spark: Workplace Violence in an Infectious Disease Hospital—A Longitudinal Study

**DOI:** 10.1155/2013/316358

**Published:** 2013-06-26

**Authors:** Nicola Magnavita

**Affiliations:** Department of Public Health, Università Cattolica del Sacro Cuore, Largo Agostino Gemelli 8, 00168 Rome, Italy

## Abstract

*Objectives. *Workplace violence (WV) is an important occupational hazard for healthcare workers (HCWs). *Methods.* A longitudinal study was carried out on HCWs from an infectious disease hospital. Work-related stress, anxiety, and depression were measured at baseline in 2003, and they were reassessed in 2005, along with the assaults that occurred in the previous year. *Results*. One-year prevalences of 6.2% and 13.9% were reported for physical and verbal aggressions, respectively. Perpetrators were mainly patients. The professional groups most frequently attacked were physicians, followed by nurses. Workers with job strain at baseline had a significant risk of being subject to aggression (OR 7.7; CI 95%, 3.3–17.9) in the following year. The relationship between job strain and subsequent WV remained significant even after correction for anxiety, depression, and other confounders. Conversely, experiencing WV was associated with a high risk of job strain and effort-reward imbalance in the following year. The final levels of anxiety and depression were predicted using regression models that included physical aggression among predictive variables. *Conclusions.* WV is the spark that sets off a problematic work situation. Effective prevention of WV can only be achieved within the framework of an overall improvement in the quality of work.

## 1. Introduction

The occurrence of workplace violence (WV) towards health care workers (HCWs) is a quite frequent and widespread phenomenon [1]. Workers employed in psychiatric or emergency services and first aid are at greater risk of violence [2]. However, no type of health care is completely exempt from the threat of violence, as it has been shown by studies on radiologists [3, 4], one of the safer, but not entirely risk-free, specialties. 

The occurrence of violence in specialized centers for infectious diseases has never been studied. These hospitals have some special features that need analyzing, because they can affect the risk of violence against staff.

The first factor concerns patients. A Canadian case-control, retrospective analysis of disruptive/aggressive behavior in hospital patients found that, compared with controls, they have a more than seven-fold increased risk of having been diagnosed with infectious diseases (OR 7.6; CI 95%, 1.4 to 41.7). The authors failed to give an interpretation of this finding [5]. It is well known, however, that people with infectious diseases may develop cognitive impairment [6], which may in turn promote aggression against staff [7].

 Many infectious diseases, such as hepatitis C [8] and HIV/AIDS [9], are currently concentrated among vulnerable populations such as injecting drug users, sex workers, and their clients. Sex workers often complain of “general hostility from public-sector providers”, “criminalization”, and “stigmatization” [10]. Whether these claims are true or not, this kind of statement is an expression of the difficult relationship with medical staff. Drug abusers, on their part, often manifest antisocial, criminal, and violent behavior [11]. A recent international study of the effects of methadone maintenance treatment programs showed that they do reduce heroin dependence, but not opioid-related crime [12]. Even after treatment, drug-dependent individuals frequently re-engage in criminal activity linked to their dependence [13]. Opioid dependence is now widely recognized as a mental disorder and has been shown to permanently alter brain function [13]. Compared with patients in general wards, patients with infectious diseases may suffer more frequently from cognitive, behavioral, and psychiatric problems and also have a criminal record, thus potentially increasing the risk of assaults against staff.

Conversely, these specialized hospitals have some general characteristics that moderate the risk of aggression or that are relevant to the type of attacks that can occur. First, according to recommendations for environmental infection control in health-care facilities [14], visitor routes are different from those used by patients and medical staff. This explains why aggression towards the staff on the part of friends, relatives, and visitors is unusual. Second, the need to isolate infectious patients often means that in each department of infectious diseases there are fewer patients than on an ordinary medical ward. However, since these patients are often in a critical condition, nurses often act in groups and not alone. Finally, the staff is partly engaged in scientific tasks and laboratory analysis, which reduces contact with patients and therefore the risk of violence from patients towards staff.

In the present study, we intend to evaluate the frequency of violence in a hospital dedicated solely to the care of patients with infectious diseases. Using a longitudinal method, we also aim to study the consequences of violence, analyzing in particular the relationship with occupational stress, anxiety, and depression. Finally, we wish to ascertain whether workers' personal characteristics, state of distress, or psychological problems may influence the occurrence of acts of violence.

## 2. Method

During their periodical medical examinations at the workplace, hospital workers were invited to respond to a questionnaire about their own occupational risks and state of health. In 2003, the questionnaire included a baseline assessment of work stress, anxiety, and depression. In 2005, workers were invited to reassess their self-perceived level of work stress, anxiety, and depression, and they were asked to describe their experience of workplace violence with reference to the previous 12 months. Complete responses were obtained both in 2003 and in 2005 from 627 workers, or 97.8% of workers called for the routine medical examination (641).

Routine medical examination is compulsory in Italy for all workers exposed to occupational risks (e.g., chemical, biological, physical, and organizational/psychosocial hazards). For many years, the workers participating in this study had been accustomed to completing questionnaires while waiting for medical examination, so they were aware that the results would be used in their interest. Although participation was not obligatory, most of the workers chose to complete the questionnaire. The Ethics Committee of the Università Cattolica del Sacro Cuore of Rome approved the study design.

### 2.1. Questionnaires

Occupational stress was measured in 2003 and 2005 using the DCS Demand/Control/Support Questionnaire derived from the longer Job Content Questionnaire [15], and, in 2005, it was used in conjunction with the Effort/Reward Questionnaire [16]. Both questionnaires were translated into Italian and validated [17]. The classic 17-item DCS Questionnaire consisted of 3 scales termed “psychological job demand”, “job control or decision latitude”, and “workplace social support”. The “Demand” scale was the sum of 5 items (e.g., D1: “Do you have to work very fast in your job?”); the “Control” scale was the sum of 6 items (e.g., C1: “Do you have the opportunity to learn new things in your work”); and the “Support” scale was the sum of 6 items (e.g., S1: “There is a calm and pleasant atmosphere where I work.”). Items were scored using a 4-point Likert scale in which the first two scales were graded from 1 = never to 4 = often, while the third scale (Support) was graded from 1 = strong disagreement to 4 = strong agreement. Validation of the translation ensured that the Italian version maintained the characteristics of the original and that the internal reliability (Cronbach's alpha) of each subscale was satisfactory (values: 0.71, 0.65, and 0.84, resp.). We followed the commonest method of obtaining a continuous variable, termed “perceived job strain”, that was obtained by dividing Demand by Control (weighted by item numbers). 

The 23-item ERI Questionnaire contained two scales: “Effort”, evaluated by 6 items (e.g., E1: “I have constant time pressure due to a heavy workload.”) and “Reward”, evaluated by 11 items (e.g., R1: “I receive the respect I deserve from my superior or equivalent person.”). Both were scored on a 5-point scale, where a value of 1 indicated no stressful experience and 5 indicated a highly stressful experience. The weighted ratio between effort and reward was calculated to quantify the degree of mismatch between effort and reward. The ERI Questionnaire also included a third scale, “Overcommitment”, which was evaluated by 6 items on a 4-point Likert scale (e.g., O3: “When I get home, I can easily relax and “switch off” work.”). It measured the set of intrinsic personal factors regarding occupational motivation and participation that enhance the effects of stress. Consequently, the score for subscale E ranged from 6 to 30 points, that for subscale R ranged from 11 to 55 points, and the subscale O score ranged from 6 to 24 points. Cronbach's alpha for the three subscales was 0.89, 0.88, and 0.89, respectively. 

Physical aggression was defined as forceful, hostile, or aggressive behavior which may or may not cause harm. Verbal (nonphysical) aggression was defined as any annoying or unpleasant act (words, attitudes, and actions) that creates a hostile work environment. 

The characteristics of incidents were studied using the Italian version of the VIF (Violent Incident Form), a validated questionnaire proposed by Arnetz for the registration of violent incidents in the health care workplace [18] and previously used in other Italian studies [19, 20]. The VIF consists of 11 clear-cut questions with binary (yes/no) responses for describing a specific incident of violent or harassing behavior directed toward a staff member. It includes domains about the perpetrator (origin, sex, age, and status), the activity that preceded the incident, the type of assault, the action taken by the victim, and the consequences of the incident. Reliability, as evaluated by the one-month test-retest Spearman-Brown split-half coefficient was, 0.91 [19].

Anxiety and depression were assessed using the Italian version [21] of the Goldberg scales [22]. This short interview, designed to be used by nonpsychiatrists, is composed of two scales of 9 binary items; a score of one is recorded against each question answered in the affirmative. Each scale provides a variable with values ranging between 0 and 9. People with anxiety scores of five or depression scores of two have a 50% chance of having a clinically important disturbance; above these scores the probability rises sharply [23]. Consequently, workers who scored five or more were classified as “anxious”, while workers who scored two or more were classified as “depressed”. The internal consistency reliability coefficient (Cronbach's alpha) value was 0.82 for the anxiety scale and 0.78 for the depression scale.

### 2.2. Statistical Analysis

Analyses began with basic descriptive statistics on the sample and crude estimates of event rates. Stress and psychiatric variables measured at the beginning and at the end of the observation period were compared using the Wilcoxon signed-rank test. The relationship between psychosocial variables and workplace violence was assessed by binary logistic regression analysis. Demand, control, support, and job strain were dichotomized at the median. Anxiety and depression scores were recorded as binary variables according to the cut-off levels of caseness. Odds ratios (ORs) and 95% confidence intervals (CIs) were calculated. 

Logistic regression analysis was used first of all to ascertain the association between stress measured in 2003 and subsequent violence. Initially, each variable was included singly in a univariate logistic regression model, and the crude odds ratio was calculated; the value was then corrected by inserting the confounders (age, gender, and profession). Finally, a multivariate regression model was built that included, as independent variables, job strain, social support, anxiety, depression, and all of the above listed demographic and occupational variables.

In a second step, logistic regression analysis was used to assess the effect of violence on work-related stress. In this case, stress measured in 2005 (demand, control, support, job strain, effort, reward, overcommitment, and effort/reward imbalance), anxiety, and depression were set as the dependent variables in separate models, while violent events (physical or nonphysical assault) were considered independent variables.

Simple linear regression with the backward-stepwise selection method was used to analyze which variables were best predictors of anxiety and depression. In two distinct models, the final level of anxiety (or depression) was taken as the dependent variable, while age, baseline anxiety (or depression), demand, control, support, effort, reward, and overcommitment were used as independent variables.

Analysis was performed using the IBM/SPSS 20.0 statistical package. 

## 3. Results

Two hundred sixty-eight male health care workers (42.7%) and 359 female workers (57.3%) were included in the study. The occupational characteristics of the population are reported in Table 1.

During the 12-month period preceding medical examination, 39 workers (6.2%) reported one episode of physical assault; nonphysical aggression with threats or other forms of verbal violence (87 cases, 13.9%) was also reported. Perpetrators were mainly patients, but some of the verbal aggression came from colleagues or superiors (16 cases, 12.7%). The attacks often occurred when the health care worker was giving the patient treatment or was discussing or responding to requests from the patient; in most cases (78, 62%), they were sudden, and the employee had no means of predicting the outbreak of violence. The health care worker was working alone in 75% of cases. Doctors were the category most prone to attacks, followed by nurses; attacks against laboratory technicians, office staff, or other professional categories were rare. Physical attacks took the form of pinching, restraining, jerking, or pushing, and it resulted in physical injury only in a minority of cases; no major episodes involving the use of a weapon were described. The immediate consequences of the attacks were anger, distress, disappointment, and helplessness. 

The psychosocial values measured at the beginning and at the end of the investigation are shown in Table 1. In the observation period, there was a slight increase in both Demand (the Wilcoxon test: *P* = 0.043; two-tailed Student's *t-*test for paired data: *P* = 0.302) and Control (the Wilcoxon test: *P* = 0.019; *t-*test: *P* = 0.039). Consequently, the resulting quotient, or job strain, was unchanged (the Wilcoxon test: *P* = 0.97; *t-*test: *P* = 0.14). Stress measured in 2005 with the ERI model indicated an imbalance between the efforts made to complete the work and the rewards received (ERI ratio > 1). Levels of anxiety and depression increased significantly during the observation period. According to the established cut-off levels, at the end of the observations, about one person in three (30.1%) had symptoms that could be diagnosed as anxiety, and one in five (21.7%) showed signs of depression.

Logistic regression analysis showed that Demand measured at baseline was a good predictor of both physical and verbal aggressions against HCWs in the following year. All stress-related variables were significant predictors of the occurrence of nonphysical attacks, and workers with job strain had a significant risk of being subject to the threat of verbal aggression (OR 7.7; CI 95%, 3.3–17.9) in the following year (Table 2). The relationship between job strain and subsequent violence remained significant even after correction for anxiety, depression, and other confounders. 

Workers who had experienced violence in the previous year were at greater risk of work-related stress in 2005. Physical aggression was significantly associated with high self-perceived demand (OR 2.1; CI 95%, 1.1–4.4), high job strain (OR 4.9; CI 95%, 2.2–11.2), high effort (OR 2.7; CI 95%, 1.3–5.5), low reward (OR 2.4; CI 95%, 1.1–5.2), high overcommitment (OR 2.0; CI 95%, 1.02–3.9), and high E/R imbalance (OR 4.3; CI 95%, 1.9–9.7). Nonphysical aggression was associated with high demand (OR 4.2; CI 95%, 2.4–7.5), low control (OR 2.8; CI 95%, 1.6–4.9), low support (OR 2.9; CI 95%, 1.7–4.9), and high job strain (OR 6.6; CI 95%, 3.6–12.0). Nonphysical aggression was also associated with high effort (OR 3.3; CI 95%, 1.9–5.5), low reward (OR 2.8; CI 95%, 1.6–4.7), high overcommitment (OR 1.6; CI 95%, 1.01–2.6), and high E/R imbalance (OR 4.1; CI 95%, 2.4–7.1). Physical aggression was also associated with a five-fold increased odds ratio for anxiety, and with a four-fold increased OR for depression. Nonphysical aggression was associated with an increased OR for both anxiety and depression. All of the above-listed relationships, although significant, were rather weak, and determination coefficients rarely exceeded 15% (Table 3).

The final level of anxiety (measured in 2005) was predicted, with good approximation (coefficient of determination: *R* ^2^ = 0.83) using a model that included the level of anxiety measured previously (in 2003), overcommitment or “intrinsic stress”, lack of social support, and experience of physical assault. Similarly, the final level of depression was predicted (coefficient of determination: *R* ^2^ = 0.76) from the previous level of depression, the overcommitment and reward scores, the lack of control over work, and experience of physical assault (Table 4).

## 4. Discussion

To our knowledge, this study is the first to effect prospective longitudinal periodic measurements of workplace violence in a hospital specialized in infectious diseases. Our study indicates that violence against health care workers is a major problem even in highly specialized hospitals similar to the one we investigated. The overall rate of physical assault (6.2%) was significantly lower than that measured in a public health care unit in the same region, where it reached almost 10% per year [2], thus placing it in the lowermost part of the intervals of prevalence observed in hospitals, where a recent review reported a range proportion of verbal abuse (22%–90%), physical threats (12%–64%), and assaults (2%–32%), [24]. However, the consequences of this phenomenon are as important as those observed in other cases.

Those who suffered from physical or verbal violence in the workplace perceived an excessive psychological burden due to their work and reported a significant increase in their self-perceived job strain and effort-reward imbalance, as well as a reduction in social support at work. The experience of aggression was also associated with increased over-commitment. Since the ERI Questionnaire was administered only once, we cannot be certain of the direction of this association. It could mean that workers who are attacked respond by obsessively increasing their commitment to work: this would be an inappropriate coping strategy which could lead to increased strain and increased risk of aggression. On the contrary, one could argue that overcommitted workers spend more time in direct contact with patients, and this provides an opportunity for greater conflict. Studies show that the chances of suffering physical violence are 7.2 and 9.0 times greater for HCWs with moderate and high patient contact, respectively, than for those with little or no contact [25]. Whatever the case is, it is evident that experiencing workplace violence is an important factor for occupational stress.

In turn, this state of distress exposes the worker to violence since we found that the presence of job strain was a good predictor of the occurrence of physical and nonphysical aggression during the ensuing year. Employees who were given an excessive workload (a condition that is becoming increasingly common due to the growing lack of financial and human resources) as well as employees who felt that they had insufficient control over their work (a common condition in work that is hierarchized and tailored to the needs of the patient) were in a condition of distress. Although this cannot be considered a clinical disorder, it inevitably hinders relations with patients, visitors, and colleagues and thus facilitates abuse. Even those who perceived a deterioration in social relationships were particularly exposed to aggression. A distressed worker's behavior might include being preoccupied with a personal matter, being distracted, neglecting a patient's early signs of violence, or being impatient when coping with a patient's problem. The patient might perceive this kind of behavior as disrespectful and might consequently assault the staff member.

Perhaps the finding that caused us most concern was the marked increase in the anxiety and depression score of the population. This is certainly a complex phenomenon, in which a number of factors—notably Italy's dramatic financial crisis and that of the health care system—play a role. However, it should be noted that the experience of physical aggression is a significant predictor of the final level of anxiety and depression. The cyclic relationship between occupational stress and violence suggests that aggression is a small telltale sign of a situation that is gradually deteriorating. Physical aggression is the spark that acts as a detonator, but the psychological damage to workers is the result of a chronic deterioration in the quality of occupational life.

The fact that physical aggression was predictive of anxiety and depression has important implications for workers' mental health. Longitudinal studies on the relationship between physical aggressions, depression, and anxiety have been conducted in married couples [26], or with reference to sexual assaults [27], but we failed to find similar studies relating to the workplace. If our findings are confirmed by further research, they will give strong support to violence prevention programs and counseling interventions for the victims of WV.

Contrary to what has been observed in other studies, in which less-experienced, poor-qualified nurses are the prevailing victims [20], in infectious diseases hospital we investigated, doctors were the category most exposed to attack. This was probably due to their decision-making role and the fact that they often worked alone with patients. In the years when the survey was conducted in this particular hospital, there was no shortage of nurses: in the morning shifts in each ward, with a number of patients between 16 and 32, were in service five nurses. On the contrary, the doctor was required to deal personally with patients, even those with problematic behaviors, not only for therapeutic or clinical issues, but also for numerous trivial complaints about dysfunctional services. This undoubtedly increased the likelihood of disputes. Many similar situations occur during medical activities in the public health service. In fact, it has been reported that the public health care system often exposes physicians to very stressful workplaces where they experience higher rates of physical violence than their counterparts in the private sector [3, 28].

As expected, on account of the special characteristics of the infectious diseases hospital, where there is no emergency department and where visitors are always separated from patients and staff, there were no reports of aggression perpetrated by visitors or relatives of patients, while visitors were responsible for about a quarter of verbal aggressions and 22% of physical aggressions against staff in a general hospital in the same geographical area [2].

The results of our study confirm the relationship between WV and stress reported in the few longitudinal studies carried out on this topic in general or psychiatric hospitals. In addition, our study sheds light on the relationship between aggression and mental health. The European Study “NEXT”, which was conducted in 10 countries, demonstrated at baseline that a high frequency of violence was associated with high levels of burnout [29]. Poor information flow and uncertainty about patient treatment, as well as intense time pressure and unsatisfactory working hours, were related to a higher frequency of violence. At followup, violence and adverse psychosocial factors independently predicted lower organizational commitment [30]. A Norwegian cohort study showed that threats and violence at work may contribute to psychological distress in nurses' aides [31]. Cross-sectional studies have principally investigated the relationship between WV and stress. A survey conducted in a psychiatric hospital in Taiwan showed that the mental state of the staff influences the frequency of attacks. A low quality of life score recorded within a period of 7 days before an event in the psychological domain is a significant predictor of WV [32]. In other words, when health care workers feel unhappy in their psychological domain, they may be more vulnerable to WV. Zampieron et al. [33] observed that nurses are at the highest risk of aggression when they are overtired, stressed, and dissatisfied with their work, and they concluded that the prevention of aggression should be based on improved work organization. Many other cross-sectional studies have focused on the consequences of violence on HCWs. Assaults are associated with high job strain [34], burnout [35], anxiety [36], depression [37, 38], post-traumatic stress disorder [39, 40], and reduced productivity [41]. WV is associated with high psychological distress, low superior and coworker support, and low interactional justice [42]. Frequent incidents combined with a lack of social support increase the probability of high stress due to aggression [43]. WV is significantly associated with lower organizational commitment and well-being [44], and poor patient outcome [4]. 

The lack of studies on infectious diseases wards prevents comparison with similar experiences in other countries. A lower risk of physical violence for health staff working with HIV/AIDS patients was reported by Jackson and Ashley in Jamaica [45], but the reported risk estimate (OR 0.19; CI 95%, 0.22–0.99) is obviously incorrect, since the confidence interval does not include the estimated odds ratio.

Limitations in the current study include the fact that participants self-reported violence and stress; future studies should therefore be based on more objective measurements. Attempts to minimize recall bias included limiting recall of violent events to the previous 12 months—an approach that has been used in previous studies [46]. To further minimize bias, during their routine medical examination, workers were interviewed about the episodes they had reported so that ambiguous or missing information could be clarified. Moreover, to minimize common method variance, we used well-known validated questionnaires that had shown good reliability. Although we attempted to exclude all possible confounders, we cannot rule out the existence of residual confounding. Nevertheless, since the present study had a higher participation rate than similar studies on the same topic, we have confidence in our results. However, the findings of this study were limited to one hospital, so caution should be used in generalizing the results of this study to include other hospitals specialized in infectious diseases.

The time elapsed from data collection and the fact that this research has not been funded demonstrate the lack of attention given to this issue by the health authorities. In 2007, the Italian Ministry of Health issued a Recommendation specifically calling for the prevention of violence in health care facilities [47], but this recommendation has been hitherto disregarded as most Italian health care institutions still lack policies, strategies, and administrative or behavioral provisions for counteracting WV. We therefore hope that this study will draw attention to this problem, and especially to the field of hospitalization in infectious diseases, which has hitherto been neglected by research. 

Our study also has some strong points. Longitudinal studies often have a high attrition rate, which may bias the results [30]. Moreover, given the sensitivity of the issue, even cross-sectional studies often have a low participation rate, ranging from 24% to 39% [43, 48–51]. On the contrary, in our study, the collection of data on the part of a physician who had a direct knowledge of the workplaces and had had a long-term relationship with the workers increased the response rate and reduced the likelihood of inappropriate responses. Furthermore, our method encouraged workers to take part in prevention by suggesting possible remedies for workplace violence.

Our study confirms that workers who are exposed to violence suffer from work-related stress and that this state of distress is associated with anxiety and depression. It also indicates that workers with job strain are more vulnerable to acts of violence. Being vulnerable to violence is not only the result of job strain, since the logistic regression equation explains less than a quarter of the total variance. There are obviously other contextual and individual factors that promote violence and further research to identify these factors would provide useful information for prevention. In order to reduce violence at the workplace, it has been suggested that HCWs, who perceive a lowering of the quality of their standard of life, should monitor their own behavior carefully; alternatively, they should consider taking a short leave of absence until they feel better [32]. However, if these proposals are adopted merely at individual level, they are likely to be ineffective or even harmful. Excessive attention to one's own job and working behavior can easily become overcommitment, and we have demonstrated that overcommitment is associated with anxiety and depression. Taking short-time absence from work is not restorative and generally causes an increase, not a decrease, in work-related stress [52, 53]. The solution to the problem must be sought in overall global strategy that involves both company management and employees in attempts to improve the overall quality of work. On the basis of our findings, we suggest that programs for preventing violence in the workplace should be accompanied by intervention to reduce occupational stress and improve workers' well-being.

## Figures and Tables

**Table 1:** Characteristics of the observed sample.

	2003	2005	
Number of workers	627		
Male workers *N* (%)	268 (42.7)		
Female workers *N* (%)	359 (57.3)		
Age, mean ± SD (years)	37.5 ± 10.4		
Length of service, mean ± SD (years)	9.9 ± 8.2		
Physicians *N* (%)	79 (12.6)		
Nurses *N* (%)	335 (53.4)		
Other^(1)^ *N* (%)	213 (34.0)		
Work-related stress variables			*P* ^(2)^
Demand, range 5–20 (mean ± SD) (s.e.)	13.4 ± 2.7(0.11)	13.5 ± 2.8 (0.11)	0.043*
Control, range 6–24 (mean ± SD) (s.e.)	17.0 ± 3.2 (0.13)	17.2 ± 3.0 (0.12)	0.019*
Support, range 6–24 (mean ± SD) (s.e.)	20.3 ± 3.2 (0.13)	20.2 ± 3.2 (0.13)	0.534 (n.s.)
Job strain (D/C rate) (mean ± SD) (s.e.)	0.99 ± 0.33 (0.01)	0.98 ± 0.30 (0.01)	0.973 (n.s.)
Effort, range 6–30 (mean ± SD)		12.6 ± 3.3	
Reward, range 11–55 (mean ± SD)		19.8 ± 6.2	
Overcommitment, range 6–24 (mean ± SD)		9.3 ± 3.0	
ERI (E/R rate)		1.2 ± 0.4	
Anxiety range 0–9 (mean ± SD)	3.3 ± 2.6	3.7 ± 2.9	0.000***
Depression range 0–9 (mean ± SD)	1.7 ± 1.8	1.9 ± 2.0	0.000***
Anxious, score 6 or more (*N*; %)	142 (22.6)	189 (30.1)	
Depressed, score 4 or more (*N*; %)	108 (17.2)	136 (21.7)	

Cases of violence in 2004-2005	Physical aggression (*N*; %)	Verbal aggression (*N*; %)	
	39 (6.2)	87 (13.9)	

Physicians (prevalence)	15 (19.0)	27 (34.2)	
Nurses (prevalence)	20 (6.0)	51 (15.2)	
Other^(1)^ (prevalence)	4 (1.9)	9 (4.2)	
*P* ^(3)^	0.000***	0.000***	

^(1)^This category includes laboratory technicians, radiology technicians, physiotherapists, ancillary personnel, blue-collar workers, and clerks.

^(2)^The Wilcoxon signed-rank test; (3): Chi-square test; *significant at *P* < 0.05; significant at *P* < 0.01; ***significant at *P* < 0.001; (n.s.): not significant.

**Table 2:** Association of psychosocial variables measured in 2003 with violence occurring in the following year. Logistic regression analysis, Odds ratios (ORs), and 95% CIs (unadjusted and adjusted for age, gender, and job).

Variable	Physical aggression (*N* = 39)	Verbal aggression (*N* = 87)
Demand		
Un.	1.25 (1.09–1.42)***	1.28 (1.16–1.41)***
Ad.^a^	1.26 (1.09–1.46)**	1.28 (1.15–1.42)***
Control		
Un.	1.05 (0.95–1.17)	0.94 (0.87–1.00)
Ad.^a^	0.96 (0.85–1.08)	0.84 (0.77–0.91)***
Support		
Un.	0.94 (0.85–1.02)	0.91 (0.85–0.97)**
Ad.^a^	0.91 (0.82–1.01)	0.89 (0.83–0.96)**
Job strain		
Un.	1.71 (0.70–4.18)	3.67 (1.99–6.76)***
Ad.^a^	4.19 (1.38–12.7)*	9.85 (4.52–21.47)***
Anxiety		
Un.	0.95 (0.83–1.08)	1.05 (0.97–1.14)
Ad.^a^	1.00 (0.87–1.15)	1.11 (1.01–1.22)*
Depression		
Un.	1.08 (0.92–1.28)	1.10 (0.98–1.24)
Ad.^a^	1.20 (1.01–1.44)*	1.19 (1.05–1.35)**

Final model^b^		
Job strain	3.25 (0.97–10.95)	7.72 (3.33–17.86)***
Nagelkerke *R* ^2^ (explained variance)	0.17	0.23

*P < 0.05; **P < 0.01; ***P < 0.001.

^a^Adjusted for age, gender, and job.

^b^Adjusted for social support, anxiety, depression, age, gender, and job.

**Table 3:** Association of workplace violence with occupational stress measured in 2005. Logistic regression analysis, Odds ratios (ORs), and 95% CIs (unadjusted and adjusted for age, gender, job, and department) and Nagelkerke *R* ^2^ (explained variance) of significant associations.

Variable	Demand^1^	Control^2^	Support^2^	Job strain^1^
Physical aggression (*N* = 39)				
Un.	2.39 (1.19–4.81)* *R* ^2^ = 0.14	1.44 (0.70–2.94)	2.10 (1.03–4.30)* *R* ^2^ = 0.01	4.29 (1.94–9.49)*** *R* ^2^ = 0.03
Ad.	2.11 (1.12–4.38)* *R* ^2^ = 0.10	1.95 (0.93–4.09)	1.95 (0.94–4.06)	4.94 (2.18–11.18)*** *R* ^2^ = 0.12
Nonphysical aggression (*N* = 87)				
Un.	4.69 (2.71–8.09)*** *R* ^2^ = 0.08	2.02 (1.19–3.43)** *R* ^2^ = 0.02	3.04 (1.80–5.16)*** *R* ^2^ = 0.04	5.59 (3.17–9.87)*** *R* ^2^ = 0.09
Ad.	4.24 (2.40–7.49)*** *R* ^2^ = 0.15	2.78 (1.59–4.87)*** *R* ^2^ = 0.08	2.87 (1.66–4.94)*** *R* ^2^ = 0.06	6.61 (3.64–12.0)*** *R* ^2^ = 0.18

	Effort^1^	Reward^2^	Overcommitment^1^	E/R imbalance^1^

Physical aggression (*N* = 39)				
Un.	2.78 (1.38–5.59)** *R* ^2^ = 0.02	2.46 (1.18–5.14)* *R* ^2^ = 0.01	2.03 (1.06–3.91)* *R* ^2^ = 0.01	4.35 (1.97–9.62)*** *R* ^2^ = 0.03
Ad.	2.69 (1.31–5.55)** *R* ^2^ = 0.07	2.44 (1.15–5.18)* *R* ^2^ = 0.02	2.00 (1.02–3.90)* *R* ^2^ = 0.02	4.33 (1.92–9.74)*** *R* ^2^ = 0.07
Nonphysical aggression (*N* = 87)				
Un.	3.39 (2.07–5.57)*** *R* ^2^ = 0.05	2.70 (1.62–4.51)*** *R* ^2^ = 0.03	1.68 (1.06–2.65)* *R* ^2^ = 0.01	4.16 (2.45–7.04)*** *R* ^2^ = 0.07
Ad.	3.28 (1.95–5.51)*** *R* ^2^ = 0.10	2.79 (1.64–4.75)*** *R* ^2^ = 0.04	1.62 (1.01–2.61)* *R* ^2^ = 0.02	4.14 (2.39–7.15)*** *R* ^2^ = 0.10

	Anxiety^1^	Depression^1^		

Physical aggression (*N* = 39)				
Un.	3.85 (1.80–8.24)*** *R* ^2^ = 0.03	3.39 (1.62–7.09)*** *R* ^2^ = 0.03		
Ad.	5.00 (2.27–11.0)*** *R* ^2^ = 0.08	4.04 (1.89–8.62)*** *R* ^2^ = 0.06		
Nonphysical aggression (*N* = 87)				
Un.	2.14 (1.34–4.33)*** *R* ^2^ = 0.02	2.32 (1.45–3.73)*** *R* ^2^ = 0.03		
Ad.	2.61 (1.60–4.30)*** *R* ^2^ = 0.07	2.66 (1.61–4.39)*** *R* ^2^ = 0.06		

^1^Higher than median value; ^2^lower than median value.

*P < 0.05; **P < 0.01; ***P < 0.001.

**Table 4:** Standardized linear regression coefficients for selected variables that have predictive value on the final level of anxiety and depression observed in health care workers. Linear regression analysis, stepwise-backward selection. Independent variables entered at step 0: age, baseline level of anxiety or depression, physical aggression, verbal aggression, demand, control, support, effort, reward, and overcommitment.

	Standardized coefficient	*t*	*P*
	Beta		
Anxiety (final level)			
(Constant)		−3.423	0.001
Anxiety^1^	0.867	50.167	0.000
Physical aggression	0.160	9.534	0.000
Support	−0.046	−2.664	0.008
Overcommitment	0.088	5.197	0.000
(*R* ^2^ = 0.83)			
Depression (final level)			
(Constant)		−1.716	0.087
Depression^1^	0.838	41.230	0.000
Physical aggression	0.084	4.204	0.000
Control	−0.041	−2.069	0.039
Reward	0.049	2.259	0.024
Overcommitment	0.050	2.279	0.023
(*R* ^2^ = 0.76)			

^1^Baseline Level.

## References

[1] Warren B (2011). Workplace violence in hospitals: safe havens no more. *Journal of Healthcare Protection Management*.

[2] Magnavita N, Heponiemi T (2012). Violence towards health care workers in a public health care facility in Italy: a repeated crosssectionalstudy. *BMC Health Services Research*.

[3] Magnavita N, Fileni A, Pescarini L, Magnavita G (2012). Violence against radiologists. I: prevalence and preventive measures. *La Radiologia Medica*.

[4] Magnavita N, Fileni A (2012). Violence against radiologists. II: psychosocial factors. *La Radiologia Medica*.

[5] Boggild AK, Heisel MJ, Links PS (2004). Social, demographic, and clinical factors related to disruptive behaviour in hospital. *Canadian Journal of Psychiatry*.

[6] Katan M, Moon YP, Paik MC, Sacco RL, Wright CB, Elkind MS (2013). Infectious burden and cognitive function: the Northern Manhattan Study. *Neurology*.

[7] Scott A, Ryan A, James I, Mitchell EA (2011). Perceptions and implications of violence from care home residents with dementia: a review and commentary. *International Journal of Older People Nursing*.

[8] Alter MJ (2002). Prevention of spread of hepatitis C. *Hepatology*.

[9] Grassly NC, Garnett GP (2005). The future of the HIV pandemic. *Bulletin of the World Health Organization*.

[10] Scorgie F, Nakato D, Harper E (2013). We are despised in the hospitals“: sex workers” experiences of accessing health care in four African countries. *Culture, Health & Sexuality*.

[11] Brooner RK, Schmidt CW, Felch LJ, Bigelow GE (1992). Antisocial behavior of intravenous drug abusers: implications for diagnosis of antisocial personality disorder. *American Journal of Psychiatry*.

[12] Mattick RP, Breen C, Kimber J, Davoli M (2009). Methadone maintenance therapy versus no opioid replacement therapy for opioid dependence. *Cochrane Database of Systematic Reviews*.

[13] Wu Z (2013). Arguments in favour of compulsory treatment of opioid dependence. *Bulletin of the World Health Organization*.

[14] Sehulster L, Chinn RYW (2003). Guidelines for environmental infection control in health-care facilities. Recommendations of CDC and the Healthcare Infection Control Practices Advisory Committee (HICPAC). *MMWR Recommendations and Reports*.

[15] Karasek RA (1979). Job demands, job decision latitude, and mental strain. Implication for job redesign. *Administrative Science Quarterly*.

[16] Siegrist J (1996). Adverse health effects of high-effort/low-reward conditions. *Journal of Occupational Health Psychology*.

[17] Magnavita N (2007). Two tools for health surveillance of job stress: the Karasek job content questionnaire and the siegrist effort reward imbalance questionnaire. *Giornale Italiano di Medicina del Lavoro ed Ergonomia*.

[18] Arnetz JE (1998). The Violent Incident Form (VIF): a practical instrument for the registration of violent incidents in the health care workplace. *Work and Stress*.

[19] Magnavita N, Heponiemi T (2011). Workplace violence against nursing students and nurses: an Italian experience. *Journal of Nursing Scholarship*.

[20] Magnavita N (2011). Violence prevention in a small-scale psychiatric unit: program planning and evaluation. *International Journal of Occupational and Environmental Health*.

[21] Magnavita N (2007). Ansia and depression at work. The aid goldberg questionnaire. *Giornale Italiano di Medicina del Lavoro ed Ergonomia*.

[22] Goldberg D, Bridges K, Duncan-Jones P, Grayson D (1988). Detecting anxiety and depression in general medical settings. *British Medical Journal*.

[23] Gann M, Corpe U, Wilson I (1990). The application of a short anxiety and depression questionnaire to oil industry staff. *Journal of the Society of Occupational Medicine*.

[24] Pompeii L, Dement J, Schoenfisch A (2013). Perpetrator, worker and workplace characteristics associated with patient and visitor perpetrated violence (Type II) on hospital workers: a review of the literature and existing occupational injury data. *Journal of Safety Research*.

[25] Canton AN, Sherman MF, Magda LA (2009). Violence, job satisfaction, and employment intentions among home healthcare registered nurses. *Home Healthcare Nurse*.

[26] Keiley MK, Keller PS, El-Sheikh M (2009). Effects of physical and verbal aggression, depression, and anxiety on drinking behavior of married partners: a prospective and retrospective longitudinal examination. *Aggressive Behavior*.

[27] Creighton CD, Jones AC (2012). Psychological profiles of adult sexual assault victims. *Journal of Forensic and Legal Medicine*.

[28] Heponiemi T, Kouvonen A, Sinervo T, Elovainio M (2013). Is the public healthcare sector a more strenuous working environment than the private sector for a physician?. *Scandinavian Journal of Public Health*.

[29] Estryn-Behar M, Van Der Eijden B, Camerino D (2008). Violence risks in nursing-results from the European “NEXT” study. *Occupational Medicine*.

[30] Camerino D, Estryn-Behar M, Conway PM, van Der Heijden BIJM, Hasselhorn H (2008). Work-related factors and violence among nursing staff in the European NEXT study: a longitudinal cohort study. *International Journal of Nursing Studies*.

[31] Eriksen W, Tambs K, Knardahl S (2006). Work factors and psychological distress in nurses' aides: a prospective cohort study. *BMC Public Health*.

[32] Chen WC, Huang CJ, Hwang JS, Chen CC (2010). The relationship of health-related quality of life to workplace physical violence against nurses by psychiatric patients. *Quality of Life Research*.

[33] Zampieron A, Galeazzo M, Turra S, Buja A (2010). Perceived aggression towards nurses: study in two Italian health institutions. *Journal of Clinical Nursing*.

[34] Shiao JS, Tseng Y, Hsieh Y, Hou J, Cheng Y, Guo YL (2010). Assaults against nurses of general and psychiatric hospitals in Taiwan. *International Archives of Occupational and Environmental Health*.

[35] Gascon S, Leiter MP, Pereira JP (2012). The role of aggressions suffered by healthcare workers as predictors of burnout. *Journal of Clinical Nursing*.

[36] Pai HC, Lee S (2011). Risk factors for workplace violence in clinical registered nurses in Taiwan. *Journal of Clinical Nursing*.

[37] Aytac S, Bozkurt V, Bayram N (2011). Workplace violence: a study of Turkish workers. *International Journal of Occupational Safety and Ergonomics*.

[38] Hansen AM, Hogh A, Persson R, Karlson B, Garde AH, Ørbæk P (2006). Bullying at work, health outcomes, and physiological stress response. *Journal of Psychosomatic Research*.

[39] Gates DM, Gillespie GL, Succop P (2011). Violence against nurses and its impact on stress and productivity. *Nursing Economics*.

[40] Laposa JM, Alden LE, Fullerton LM (2003). Work stress and posttraumatic stress disorder in ED nurses/personnel. *Journal of Emergency Nursing*.

[41] Kowalenko T, Gates D, Gillespie GL, Succop P, Mentzel TK (2013). Prospective study of violence against ED workers. *American Journal of Emergency Medicine*.

[42] Tsuno K, Kawakami N, Inoue A, Abe K (2010). Measuring workplace bullying: reliability and validity of the Japanese version of the negative acts questionnaire. *Journal of Occupational Health*.

[43] Franz S, Zeh A, Schablon A, Kuhnert S, Nienhaus A (2010). Aggression and violence against health care workers in Germany-a cross sectional retrospective survey. *BMC Health Services Research*.

[44] Rodwell J, Demir D, Parris M, Steane P, Noblet A (2012). The impact of bullying on health care administration staff: reduced commitment beyond the influences of negative affectivity. *Health Care Management Review*.

[45] Jackson M, Ashley D (2005). Physical and psychological violence in Jamaica's health sector. *Revista Panamericana de Salud Publica*.

[46] Gerberich SG, Church TR, McGovern PM (2004). An epidemiological study of the magnitude and consequences of work related violence: the Minnesota Nurses' Study. *Occupational and Environmental Medicine*.

[47] Ministero della Salute Recommendation to prevent acts of violence against health workers. http://www.ministerosalute.it/imgs/C_17_pubblicazioni_721_allegato.pdf.

[48] Gascón S, Martínez-Jarreta B, Fabricio González-Andrade J, Ángel Santed M, Casalod Y, Ángeles Rueda M (2009). Aggression towards health care workers in Spain: a multi-facility study to evaluate the distribution of growing violence among professionals, health facilities and departments. *International Journal of Occupational and Environmental Health*.

[49] Arnetz JE, Aranyos D, Ager J, Upfal MJ (2011). Worker-on-worker violence among hospital employees. *International Journal of Occupational and Environmental Health*.

[50] Hodgson MJ, Reed R, Craig T (2004). Violence in healthcare facilities: lessons from the Veterans Health Administration. *Journal of Occupational and Environmental Medicine*.

[51] Winstanley S, Whittington R (2004). Aggression towards health care staff in a UK general hospital: variation among professions and departments. *Journal of Clinical Nursing*.

[52] Johns G, Cooper CL, Barling J (2008). Absenteeism and presenteeism. Not at work or not working well. *The Sage Handbook of Organizational Behavior*.

[53] Magnavita N, Garbarino S (2013). Is absence related to work stress? A repeated cross-sectional study on a special police forc. *American Journal of Industrial Medicine*.

